# Specimen-specific differences in clinical metagenomic sequencing reporting patterns in hospitalized patients: a single-center retrospective observational study

**DOI:** 10.3389/fcimb.2026.1823283

**Published:** 2026-05-20

**Authors:** Jian-min Ren, Xiao-yao Zhang, Xiao-peng Liu, Li-hong Pei, Wei-pin Jiang, Xiao-mei Zhang, Hui Ding, Jian-sheng Huang

**Affiliations:** Department of Clinical Laboratory, The Fifth Affiliated Hospital of Wenzhou Medical University (Lishui Municipal Central Hospital), Lishui, Zhejiang, China

**Keywords:** bronchoalveolar lavage fluid, cerebrospinal fluid, clinical metagenomics, metagenomic next-generation sequencing, mixed detection, reporting patterns

## Abstract

Clinical metagenomic next-generation sequencing (mNGS) is increasingly used in hospitalized patients, but finalized reporting patterns vary across specimen types in routine practice. We conducted a single-center retrospective observational study using routine clinical mNGS data from January 1, 2024, to December 31, 2025. A specimen-specific first-order design retained only the first eligible mNGS order per patient within each specimen category during the study window. Orders were grouped as bronchoalveolar lavage fluid (BALF), blood, cerebrospinal fluid (CSF), and tissue for primary comparisons; heterogeneous “Other” specimens were described separately. The primary endpoint was report-interpreted any-positive at the order level. We summarized specimen-specific report-interpreted positivity, pathogen-group detection, the most frequently reported organisms ranked by order-level report presence, and mixed detections among positive orders. ICU-associated analyses were included as contextual descriptive stratification only. The cohort included DNA-only orders and a subset of PMseq-RNA-tested orders; RNA virus analyses were restricted to PMseq-RNA-tested orders, and DNA-only orders were treated as not tested for RNA virus fields. Among 1, 981 included specimen-specific first orders, BALF accounted for 973, blood 473, CSF 240, and tissue 122. Report-interpreted any-positive differed by specimen type, with BALF highest (876/973, 90.0%; 95% CI, 88.0–91.8%), followed by tissue (95/122, 77.9%; 95% CI, 69.7–84.3%), blood (343/473, 72.5%; 95% CI, 68.3–76.3%), and CSF (63/240, 26.2%; 95% CI, 21.1–32.2%). Among positive orders, at least 2 distinct standardized pathogens were reported in 672/876 BALF orders (76.7%), 182/343 blood orders (53.1%), 39/95 tissue orders (41.1%), and 8/63 CSF orders (12.7%). Across the four primary specimen groups, the most frequently reported organisms included Epstein-Barr virus (n = 485), Candida albicans (n = 285), and cytomegalovirus (n = 262), together with Klebsiella pneumoniae and Acinetobacter baumannii; these rankings reflect report-level frequency rather than adjudicated pathogenic roles, particularly for latency- or reactivation-prone viruses. Of included orders, 277 (14.0%) underwent PMseq-RNA testing. These findings characterize specimen-specific differences in clinical mNGS reporting patterns and provide a specimen-context-aware reference for interpreting routine inpatient reports.

## 1 Introduction

Metagenomic next-generation sequencing (mNGS) is increasingly used in routine clinical practice for pathogen detection across different specimen types and patient populations (Chiu and Miller, 2019; Gu et al., 2019). Unlike single-target assays, mNGS can detect bacterial, fungal, viral, and selected atypical pathogens in the same specimen (Chiu and Miller, 2019; Gu et al., 2019). This is particularly relevant in critically ill or immunocompromised patients, in whom the differential diagnosis is broad and prior antimicrobial exposure is common (Gu et al., 2019; Wilson et al., 2019).

Interpretation of real-world mNGS results is affected by sampling strategy, specimen background (e.g., commensal or colonizing microbiota), and laboratory reporting rules (Chiu and Miller, 2019; Gu et al., 2019). In addition, contamination from reagents and laboratory processes may produce background signals and complicate interpretation, supporting the use of report-aware workflows and reporting conventions (Schlaberg et al., 2017; Salter et al., 2014).

Many published clinical mNGS studies remain centered on a single specimen type or a narrowly defined clinical setting, which limits comparison across anatomical sites and testing pathways. For example, recent studies have focused on bronchoalveolar lavage fluid in pneumonia or respiratory intensive care settings, whereas other high-impact work has concentrated on central nervous system infections and cerebrospinal fluid-based evaluation (Han et al., 2024; Zhang et al., 2024; Benoit et al., 2024). In parallel, prospective observational studies have evaluated clinical metagenomics in routine infectious disease diagnostics, further supporting its growing role in practice, but such studies were not designed primarily to describe finalized report patterns across specimen types in hospitalized patients (Fourgeaud et al., 2024). As a result, the contribution of pathogen groups and the frequency of mixed detections may still be difficult to compare across specimen types in routine inpatient care.

Accordingly, an important remaining gap is not only how mNGS performs within individual specimen-specific clinical scenarios, but also how finalized clinical reports differ across specimen types under routine laboratory interpretation. We therefore conducted a single-center retrospective observational study to characterize specimen-specific differences in clinical mNGS reporting patterns in hospitalized patients from January 1, 2024, to December 31, 2025. Using specimen-specific first-order cohorts and prespecified pathogen-name standardization, we described report-interpreted positivity, pathogen-group detection, frequently reported organisms, and mixed detections across BALF, blood, CSF, and tissue. ICU-associated analyses were included as an exploratory, contextual, proxy-based descriptive stratification rather than as a primary analytic objective.

## 2 Materials and methods

### 2.1 Study design, setting, and time window

We conducted a single-center retrospective observational study using routine clinical metagenomic next-generation sequencing (mNGS) data from Lishui Municipal Central Hospital, Lishui, China. Orders were generated predominantly in the inpatient setting as part of routine care, and analyses were performed at the order level based on laboratory reporting outputs. The prespecified analysis window was January 1, 2024, through December 31, 2025, defined by the specimen submission timestamp recorded in the laboratory information system (LIS). The study was designed to describe real-world testing outputs and reporting patterns rather than to estimate diagnostic accuracy against a single reference standard.

### 2.2 Data sources and variables

Order-level mNGS data were extracted from the LIS, including specimen submission timestamp, requesting department, specimen type, and finalized report interpretation (positive/negative). When available, ancillary laboratory and clinical fields, including white blood cell count (WBC), C-reactive protein (CRP), procalcitonin (PCT), conventional microbiology fields for culture/microscopy/identification, and anti-infective medication documentation, were obtained from the LIS and/or the electronic medical record (EMR) for descriptive characterization. For laboratory markers (WBC, CRP, PCT), analyses used available values without imputation; availability and missingness were summarized descriptively, and denominators are reported where applicable. For contextual description only, free-text clinical diagnosis and recorded anti-infective fields were further grouped into broad rule-based categories and summarized by specimen group (**Supplementary Tables S5, S6**).

### 2.3 Specimen-specific first-order cohorts

To reduce inflation from repeat testing while preserving specimen-specific comparisons, de-duplication was performed within each specimen category. For each patient and specimen category, only the first eligible mNGS order during the analysis window was retained. The earliest order was defined by the specimen submission timestamp. If multiple orders from the same patient within the same specimen category shared the same submission timestamp, a deterministic prespecified tie-breaking rule was applied: the order with the earliest LIS order identifier (chronological sequence) was retained. A patient could therefore contribute to more than one specimen-specific cohort, but only once within each specimen category. The unit of analysis was the included de-duplicated mNGS order.

### 2.4 Specimen grouping and analysis scope

**Figure 1:**
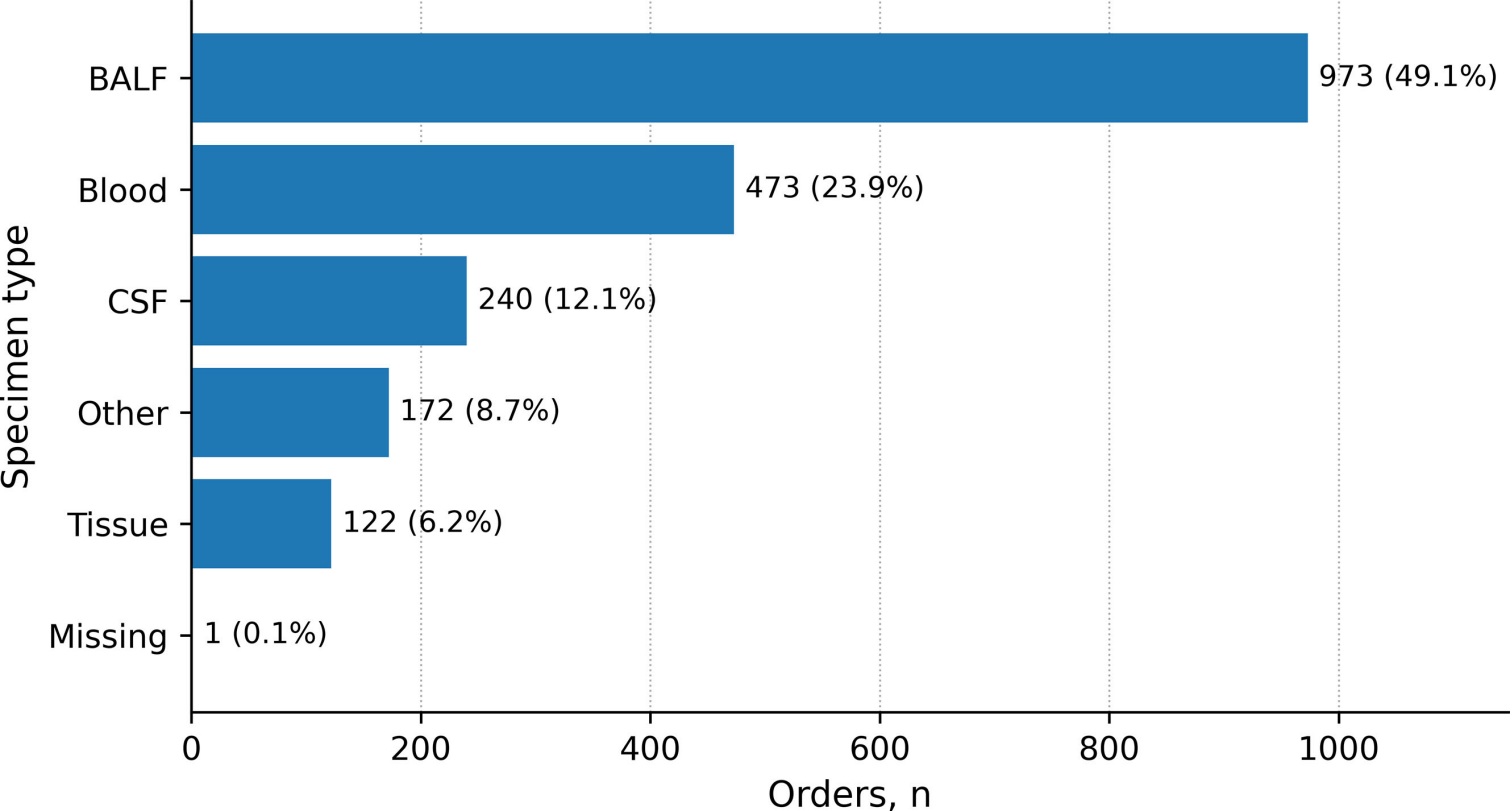
Specimen-type distribution (included specimen-specific first orders, 2024–2025). Orders were de-duplicated within each specimen category by retaining the first eligible mNGS order per patient during the study window. Specimens were grouped as BALF, blood, CSF, tissue, Other, and Missing; percentages were calculated using all included orders as the denominator. Other and Missing categories are shown for completeness and were excluded from between-group comparisons.

Specimens were grouped into four prespecified categories for between-group comparisons based on the LIS specimen-type field: bronchoalveolar lavage fluid (BALF), blood, cerebrospinal fluid (CSF), and tissue. “Tissue” comprised solid-tissue and biopsy-type specimens as labeled in the LIS. All other specimen types were categorized as “Other” and summarized descriptively; they were excluded from between-group comparisons because of heterogeneity and sparse counts. Records with missing specimen type were retained for overall description but excluded from between-group comparisons. For completeness, “Other” and “Missing” categories are shown in the overall specimen distribution figure (**Figure 1**) but were not included in comparative analyses. Overall summaries pooled the included specimen-specific first orders across specimen categories; therefore, the overall denominator reflects included orders rather than unique patients.

### 2.5 mNGS workflow, bioinformatics, report interpretation, and pathogen handling

mNGS testing followed the routine PMseq laboratory workflow used during the study period, with specimen-type-specific preprocessing and nucleic-acid extraction performed according to standardized procedures. DNA and RNA libraries were prepared using the PMseq Pathogen Sequencing Kit (BGI) following the manufacturer’s standard short-read library preparation protocol. Library quality control was performed using an Agilent 2100 system. Qualified libraries were pooled, converted to DNA nanoballs (DNBs), and sequenced on the MGISEQ-2000 platform (MGI Tech) using single-end short reads (SE75), targeting approximately 80 million reads per sample.

Bioinformatic processing was performed using the PMseq analytic framework. Reads passing quality filtering were generated after removal of low-quality reads, followed by computational subtraction of human host sequences mapped to the human reference genome hg19 using Burrows–Wheeler Alignment (BWA). Remaining reads were filtered for low-complexity sequences and taxonomically classified by alignment to the Pathogens Metagenomics Database (PMDB), which includes bacterial, fungal, viral, and parasitic references. Reference sequences were obtained from NCBI genomes.

Eligible records required a final, signed mNGS report. Failed or invalid runs without a finalized report were excluded. Orders lacking a finalized interpreted positive/negative outcome (e.g., unresolved QC failure) were excluded from analyses requiring a binary endpoint. All primary endpoints were anchored to the finalized clinical report. Specifically, “any-positive” was defined at the order level as the presence of at least 1 organism interpreted as detected/positive in the finalized report, without applying any additional *post hoc* research thresholds. Accordingly, specimen-specific positivity analyses reflected finalized clinical reporting outputs rather than re-thresholded analytical calls. The present analysis was not designed to retrospectively reconstruct organism- or specimen-specific analytical positivity thresholds across the cohort.

Organism calls were evaluated against run controls and an internal background organism list within the PMseq/PMDB framework; organisms considered consistent with background or contamination were not reported as positives in the finalized report. The laboratory applied organism- and specimen-context-aware reporting rules according to SOPs. Under the routine sign-out workflow, an initial reviewer performed the primary report check and draft interpretation, and a second qualified reviewer performed sequential verification and final sign-out; this was a sequential two-person sign-out workflow rather than a parallel independent review. For study auditability, we documented the report-interpretation and organism-handling rules relevant to endpoint construction, including eligibility, background/contamination handling, name harmonization, and class mapping. A functional summary of the PMseq report interpretation framework and endpoint anchoring is provided in **Supplementary Table 1**, and the corresponding dictionaries/lookup tables are provided in the **Supplementary Materials**.

### 2.6 Pathogen-class outcomes, composition, and mixed detections

Standardized pathogens (pathogen_name_std) were mapped to prespecified pathogen classes using a curated lookup table (pathogen_name_std → class), including bacteria, fungi, DNA viruses, RNA viruses, Mycoplasma/Chlamydia, parasites, and Mycobacterium tuberculosis complex (MTBC; “TB”). The mapping table was harmonized for synonymous labels and is provided for auditability in the Supplementary Tables (**Supplementary Tables S2–S4**).

For each pathogen class, an order was considered class-positive if at least 1 standardized organism assigned to that class was interpreted as detected/positive in the finalized report. For RNA-virus endpoints, denominators were restricted to PMseq-RNA-tested orders; DNA-only orders were coded as “not tested” for RNA-virus fields.

Composition analyses were restricted to mNGS-positive orders. Pathogen entries were counted after within-order de-duplication at the standardized-pathogen level, such that each order contributed at most one entry for a given pathogen_name_std. Mixed detection was prespecified as an order-level descriptive endpoint among mNGS-positive orders and was defined as the presence of at least 2 distinct standardized pathogens within the same finalized positive report; an additional threshold of at least 3 distinct standardized pathogens was summarized separately. All mixed-detection analyses used mNGS-positive orders as the denominator.

### 2.7 ICU and immunocompromised stratifications

ICU-associated status was defined as an order-level care-setting proxy using the requesting department. Orders requested from departments whose names matched prespecified critical-care keywords (e.g., “ICU”) were classified as ICU-associated services; all others were classified as non-ICU-associated services. Immunocompromised status was defined using a diagnosis-text proxy based on prespecified keywords indicating transplantation, chemotherapy or hematologic malignancy, HIV/AIDS, primary immunodeficiency, immunosuppressive therapy, or prolonged systemic corticosteroid use. Keyword matching used case-insensitive substring and/or regular-expression matching on the requesting-department field (ICU proxy) and diagnosis-text field (immunocompromised proxy). Full keyword dictionaries, exclusion patterns, and matching rules are provided in the **Supplementary Methods**. These proxies were used for exploratory descriptive stratification only and were not intended to support validated clinical subgroup inference or redefine primary endpoints.

### 2.8 Statistical analysis

Analyses were framed as descriptive estimation rather than hypothesis testing. Categorical variables are summarized as counts and proportions, with 95% confidence intervals (CIs) reported for prespecified key proportions, and continuous variables are summarized as medians with interquartile ranges (IQRs). Confidence intervals for proportions were calculated using the Wilson method. Endpoint-specific denominators were prespecified and applied consistently at the de-duplicated order level within specimen-specific first-order cohorts, including (i) report-interpreted any-positive by specimen group, (ii) pathogen-class presence by specimen group, (iii) Top10 pathogen rankings by order-level report presence, (iv) pathogen-class composition among positive orders using within-order de-duplicated pathogen entries, and (v) mixed detections (≥2/≥3 distinct standardized pathogens) among positive orders. Key proportions selected for CI reporting included any-positive by specimen group, mixed detections (≥2 and ≥3) among mNGS-positive orders by specimen group, and selected ICU-associated versus non-ICU-associated order-level comparisons. Because the primary objective was reporting-oriented description rather than hypothesis testing, no between-group inferential tests were prespecified and P values are not reported. Analyses were performed using R (version 4.5.2) and Python (version 3.12).

### 2.9 Ethics statement

This retrospective real-world study was approved by the Ethics Committee of Lishui Municipal Central Hospital (approval No. 2026(I)-077-01). Only existing clinical data were used, with no additional interventions. All patient data were anonymized before analysis. The requirement for informed consent was waived by the ethics committee.

## 3 Results

### 3.1 Cohort and specimen distribution

After application of the prespecified time window and de-duplication within each specimen category, 1, 981 included orders were retained for overall description. The four prespecified specimen groups comprised 1, 808 included orders and were used for between-group comparisons. Across all included orders, specimen distribution was BALF 973 (49.1%), blood 473 (23.9%), CSF 240 (12.1%), tissue 122 (6.2%), Other 172 (8.7%), and Missing 1 (0.1%) (**Figure 1**). Contextual characteristics, including ICU-associated services, immunocompromised proxy status, recorded anti-infective medication documentation, and selected laboratory markers, are summarized in **Table 1**. Broad diagnosis-text categories and grouped anti-infective fields also differed across specimen groups, further indicating distinct routine-care testing contexts (**Supplementary Tables S5, S6**).

**Table 1:** Contextual characteristics of included specimen-specific first orders by specimen group, 2024–2025.

Variable	Overall	BALF	Blood	CSF	Tissue
N (orders)	1981	973	473	240	122
ICU-associated status, n (%)	492 (24.8%)	203 (20.9%)	182 (38.5%)	49 (20.4%)	1 (0.8%)
Immunocompromised proxy status, n (%)	75 (3.8%)	31 (3.2%)	29 (6.1%)	6 (2.5%)	1 (0.8%)
Recorded anti-infective medication documentation, n (%)	1560 (78.7%)	759 (78.0%)	421 (89.0%)	165 (68.8%)	71 (58.2%)
CRP, median (IQR)	42.52 (9.69–106.80)	37.15 (8.76–92.38)	84.07 (36.55–163.38)	7.11 (0.74–23.54)	19.56 (7.51–53.65)
PCT, median (IQR)	0.18 (0.05–1.06)	0.12 (0.04–0.53)	0.93 (0.22–5.49)	0.07 (0.04–0.18)	0.05 (0.04–0.11)
Blood WBC, median (IQR)	7.70 (5.30–11.40)	7.40 (5.30–10.90)	8.32 (4.30–13.35)	8.10 (6.30–10.90)	6.75 (5.30–8.80)

Time window: January 1, 2024 to December 31, 2025, defined by the laboratory information system (LIS) specimen submission timestamp. Unit of analysis: included specimen-specific first eligible mNGS orders, defined as the first eligible order per patient within each specimen category during the study window. Accordingly, a patient could contribute to more than one specimen-specific cohort, and the overall column reflects pooled included orders rather than unique patients. Between-group summaries are restricted to the four prespecified specimen groups (BALF, blood, CSF, and tissue); Other and Missing specimen types were summarized descriptively only and were not included in comparative analyses. ICU-associated status was defined using a requesting-department proxy, and immunocompromised status was defined using a diagnosis-text proxy based on prespecified rules. Anti-infective medication documentation reflects recorded fields available from the LIS and/or EMR and is presented for contextual description only. Laboratory markers are summarized using available values only, without imputation; denominators may therefore vary by variable. Detailed diagnosis-text categories and grouped anti-infective fields are provided in **Supplementary Tables S5, S6**.

### 3.2 Specimen-specific mNGS positivity based on finalized reports

**Figure 2:**
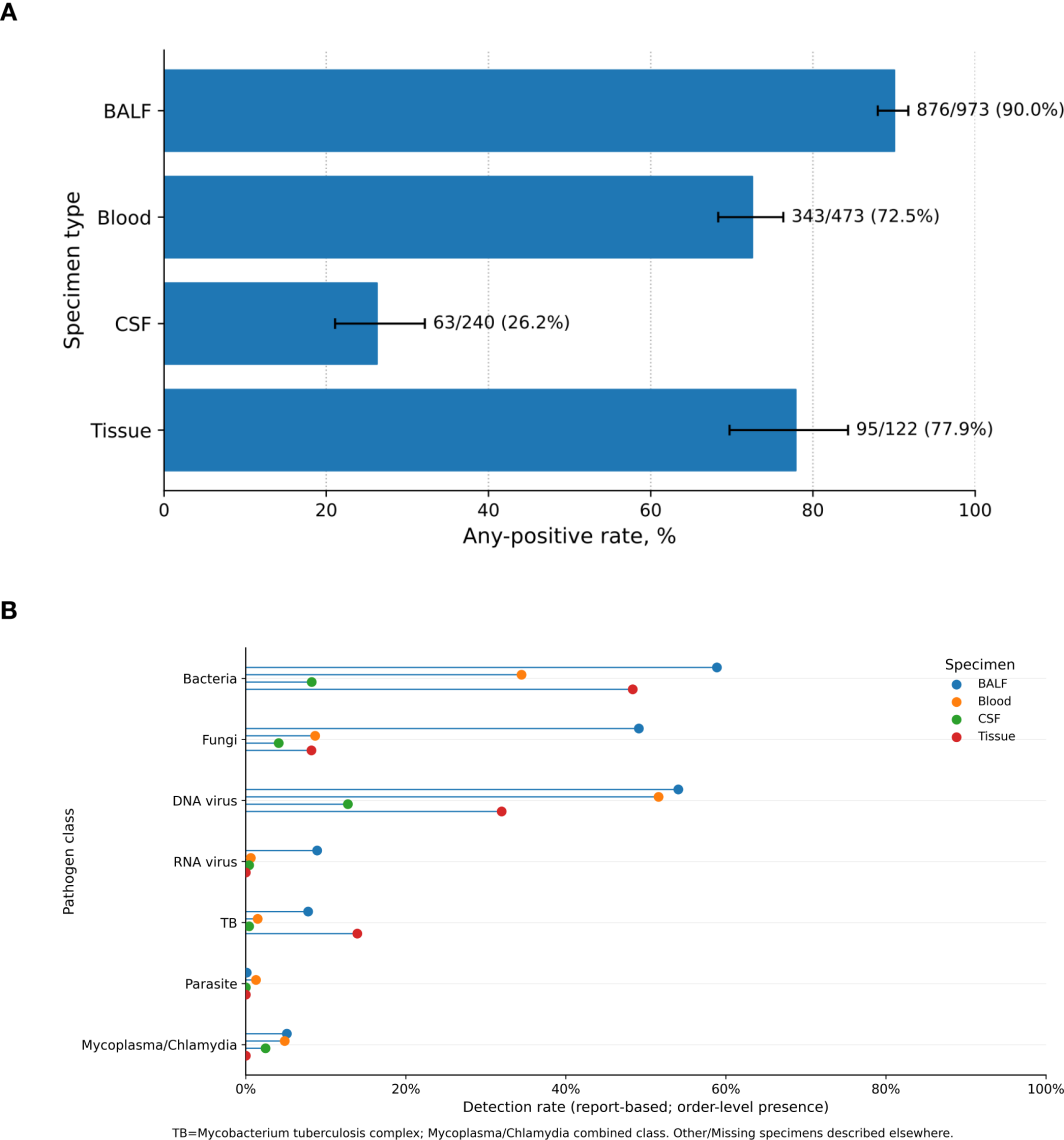
Report-interpreted any-positive rate by specimen group (2024–2025). **(A)** Report-interpreted any-positive rate by specimen group (BALF, blood, CSF, and tissue) among included specimen-specific first orders during 2024–2025. Any-positive was defined as ≥1 organism interpreted as detected/positive in the finalized report at the order level. Bars indicate the point estimate for each specimen group, labels show positive orders/total orders (%), and error bars indicate 95% confidence intervals for the corresponding proportions. **(B)** Pathogen-class detection rates across specimen groups among included specimen-specific first orders, 2024–2025. **(B)** The lollipop plot summarizes report-based, order-level detection rates of major pathogen classes by specimen group (BALF, blood, CSF, and tissue) among included specimen-specific first orders during January 1, 2024 to December 31, 2025. For each order, a pathogen class was coded as present if at least one standardized pathogen mapped to that class was reported as detected/positive in the finalized report. Percentages represent the proportion of included orders positive for each pathogen class within each specimen group. TB denotes the *Mycobacterium tuberculosis complex* (MTBC), and Mycoplasma/Chlamydia is shown as a combined class. Other and Missing specimen types were excluded from between-group comparisons. Denominators for the four primary specimen groups were BALF 973, blood 473, CSF 240, and tissue 122.

Report-interpreted any-positive rates differed markedly by specimen group (**Figure 2A**). BALF showed the highest positivity, with 876/973 orders positive (90.0%; 95% CI, 88.0–91.8%), followed by tissue (95/122, 77.9%; 95% CI, 69.7–84.3%) and blood (343/473, 72.5%; 95% CI, 68.3–76.3%). CSF had a substantially lower positivity rate (63/240, 26.2%; 95% CI, 21.1–32.2%). These estimates reflect report-interpreted order-level positivity within the prespecified specimen groups rather than adjudicated infection status.

### 3.3 Pathogen-group detection patterns and composition

**Figure 3:**
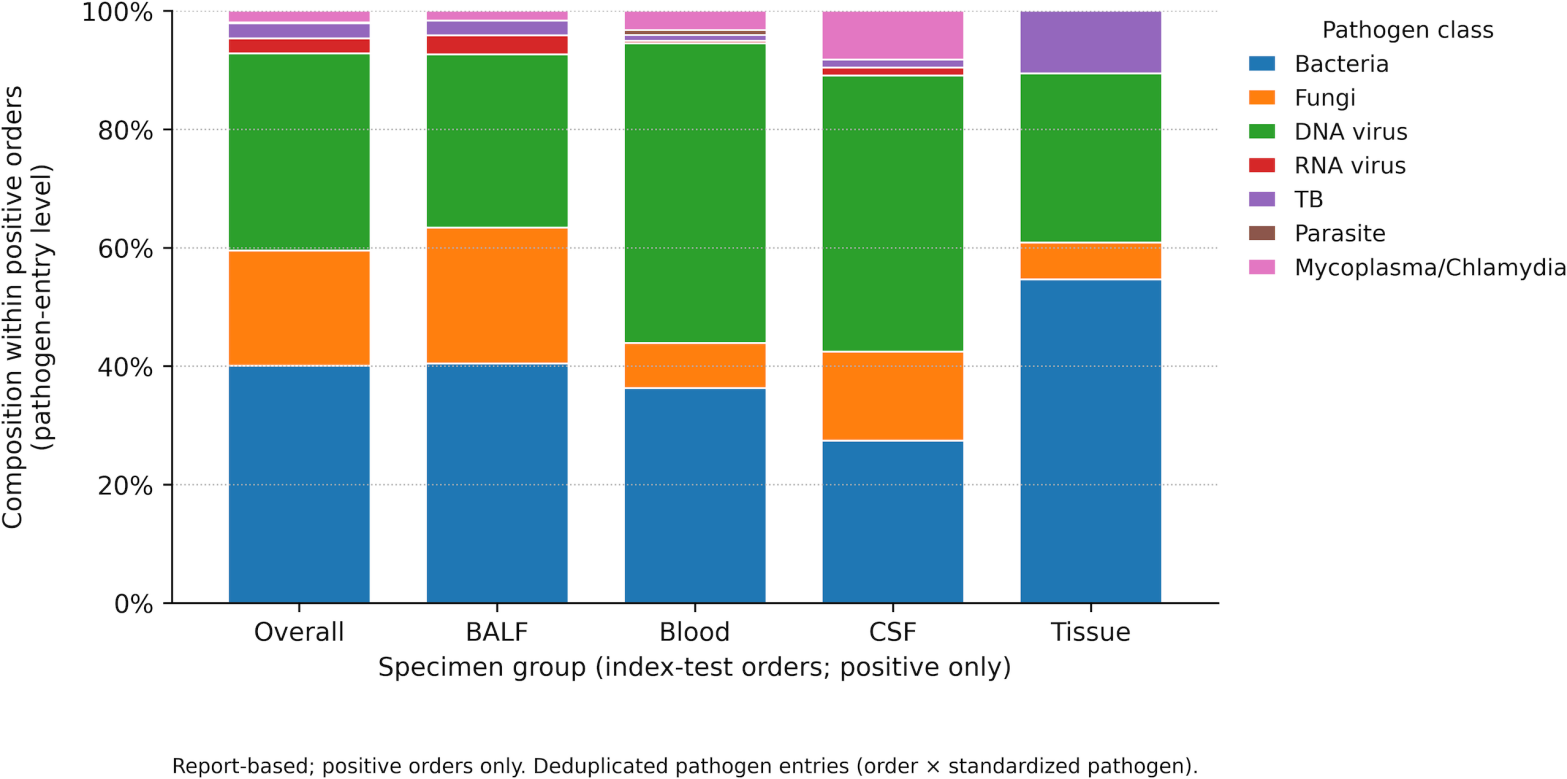
Pathogen-class composition within mNGS-positive included specimen-specific first orders by specimen group, 2024–2025. This 100% stacked bar chart summarizes pathogen-class composition among report-based mNGS-positive included orders (January 1, 2024–December 31, 2025) by specimen group (BALF, blood, CSF, and tissue), with a pooled overall column for reference. Composition was calculated at the de-duplicated pathogen-entry level (order × standardized pathogen); percentages within each specimen group sum to 100%. Classes include Bacteria, Fungi, DNA viruses, RNA viruses, Mycobacterium tuberculosis complex (MTBC; TB), Parasites, and Mycoplasma/Chlamydia. Other and Missing specimen types were excluded from between-group comparisons.

Order-level reported detection patterns by pathogen group varied across specimen types (**Figure 2B**). These summaries describe report-level organism-group presence rather than adjudicated etiologic significance, particularly for viruses that may reflect latent infection, reactivation, or other noncausal nucleic acid detection. In BALF, bacteria (59.0%), DNA viruses (54.2%), and fungi (49.2%) were commonly reported. In blood, DNA viruses were reported in 51.6% of orders, whereas bacteria were reported in 34.5%; fungal detection was less frequent (8.7%). In CSF, detection rates were lower across groups, including DNA viruses (12.9%) and bacteria (8.3%). In tissue, bacteria were reported in 48.4% of orders, and Mycobacterium tuberculosis complex detections accounted for 13.9% (**Figure 2B**). Among mNGS-positive orders, the composition of reported pathogen entries also differed by specimen type (**Figure 3**). In BALF, bacterial entries accounted for approximately 40.4% of positive-pathogen rows, DNA viruses 29.2%, and fungi 23.0%. In blood, DNA viruses accounted for 50.6% of positive-pathogen rows, followed by bacteria (36.3%). In CSF, DNA viruses accounted for 46.6% of positive-pathogen rows, followed by bacteria (27.4%) and fungi (15.1%). In tissue, bacterial entries predominated (54.7%), with DNA viruses (28.6%) and TB (10.6%) also contributing (**Figure 3**). The prominent representation of DNA viruses in blood and CSF should therefore be interpreted as a report-level pattern and should not be directly equated with active causal infection.

### 3.4 Most frequently reported pathogens (top 10)

**Figure 4:**
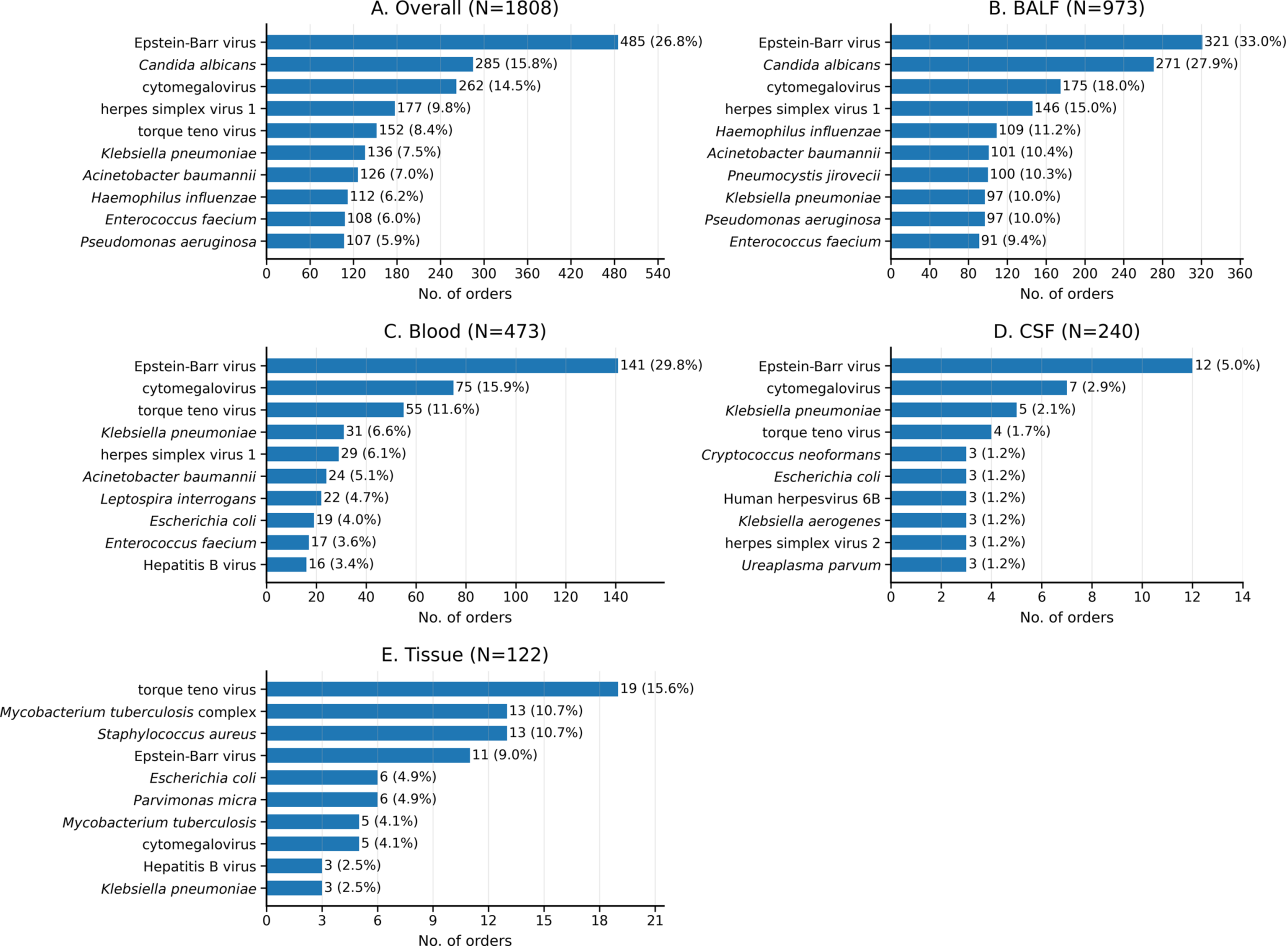
Top 10 pathogens by order-level report presence overall and by specimen group among included specimen-specific first orders, 2024–2025. Panels **(A–E)** display the Top 10 standardized pathogens ranked by order-level report presence for the pooled cohort of the four primary specimen groups **(A)** and separately for BALF **(B)**, blood **(C)**, CSF **(D)**, and tissue **(E)**. Analyses used report-interpreted results from January 1, 2024 to December 31, 2025. Within each panel, bars indicate the number of included orders in which the pathogen was reported, and labels show n (% of orders within that panel). Pathogen names were standardized using a prespecified mapping, and duplicate entries for the same standardized pathogen within an order were collapsed before counting. To preserve readability across panels with substantially different count ranges, each panel uses its own x-axis range; bar lengths should therefore be interpreted within panel rather than compared directly across panels. Orders with Other or Missing specimen types were summarized descriptively and are not shown. Bacterial and fungal binomials are shown in italics.

Across the four primary specimen groups, the Top 10 organisms ranked by order-level report presence reflected reporting frequency rather than adjudicated pathogenic roles (**Figure 4**). The most frequently reported organisms overall were Epstein–Barr virus (n = 485), *Candida albicans* (n = 285), cytomegalovirus (n = 262), herpes simplex virus 1 (n = 177), torque teno virus (n = 152), *Klebsiella pneumoniae* (n = 136), *Acinetobacter baumannii* (n = 126), *Haemophilus influenzae* (n = 112), *Enterococcus faecium* (n = 108), and *Pseudomonas aeruginosa* (n = 107). Specimen-specific Top 10 panels are shown in **Figure 4**. For latency- or reactivation-prone viruses such as Epstein–Barr virus, cytomegalovirus, and herpes simplex virus 1, frequent report presence should not be equated with proven disease causation. Torque teno virus should likewise be interpreted cautiously as a report-level signal rather than a standalone indicator of pathogenicity.

### 3.5 Mixed detections and read-count summaries

**Figure 5:**
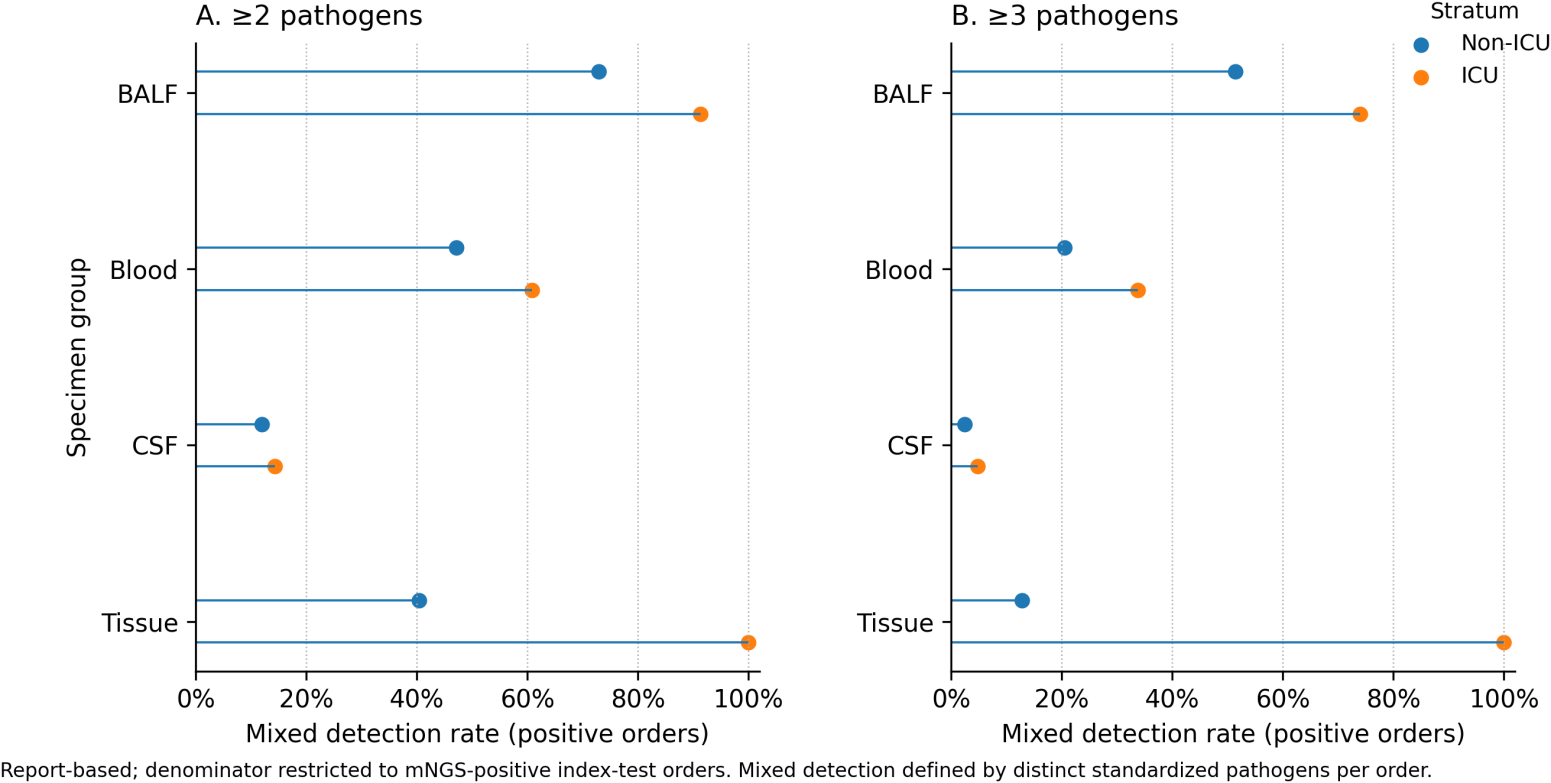
Mixed pathogen detection (≥2 and ≥3) by specimen group among mNGS-positive included orders, stratified by ICU-associated status, 2024–2025. **(A, B)** show mixed-detection frequencies among mNGS-positive included orders by specimen group (BALF, blood, CSF, and tissue) and ICU-associated status (non-ICU vs ICU). ICU-associated status was defined using a requesting-department proxy and used as an order-level care-setting attribute. Mixed detection was defined as the presence of at least 2 distinct standardized pathogens in panel **(A)** and at least 3 distinct standardized pathogens in panel **(B)** within the same finalized positive report. Percentages were calculated within each stratum using mNGS-positive included orders as the denominator. Stratum sizes were BALF, 690 (non-ICU) and 184 (ICU); blood, 195 and 148; CSF, 42 and 21; and tissue, 94 and 1.

**Figure 6:**
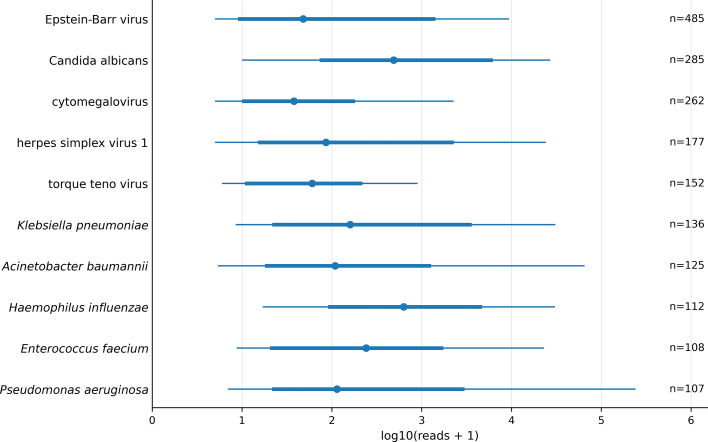
Read count distribution for the top pathogens among mNGS-positive included orders, 2024–2025. Read-count distributions for the overall Top 10 pathogens among mNGS-positive included orders, 2024–2025. Top 10 pathogens were defined by order-level report presence in the pooled cohort of the four primary specimen groups (BALF, blood, CSF, and tissue). Read-count distributions were summarized using report-interpreted results from January 1, 2024 to December 31, 2025 and were restricted to these four specimen groups. Pathogen names were standardized using a prespecified mapping, and duplicate entries for the same standardized pathogen within an order were collapsed prior to summarization. Reads are shown on the log10(reads + 1) scale. For each pathogen, the dot indicates the median, the thick segment indicates the interquartile range (P25–P75), and the thin line indicates P10–P90. The value n denotes the number of pathogen entries contributing to the distribution. Bacterial binomials are shown in italics.

Mixed detection, defined as the presence of at least 2 distinct standardized pathogens within the same finalized positive report, was common but varied substantially across specimen groups (**Figure 5**). Among mNGS-positive orders, detection of ≥2 distinct standardized pathogens was observed in BALF 672/876 (76.7%; 95% CI, 73.8–79.4%), blood 182/343 (53.1%; 95% CI, 47.8–58.3%), tissue 39/95 (41.1%; 95% CI, 31.7–51.1%), and CSF 8/63 (12.7%; 95% CI, 6.6–23.1%). Detection of ≥3 pathogens was also most frequent in BALF 492/876 (56.2%; 95% CI, 52.9–59.4%), followed by blood 90/343 (26.2%; 95% CI, 21.9–31.1%), tissue 13/95 (13.7%; 95% CI, 8.2–22.0%), and CSF 2/63 (3.2%; 95% CI, 0.9–10.9%). The wider intervals for CSF and, to a lesser extent, tissue reflect smaller positive-order denominators in these groups. Mixed detection stratified by immunocompromised proxy status is shown in **Supplementary Figure 1**. Read-count distributions for Top 10 pathogens were summarized descriptively by specimen type (**Figure 6**), with entry-level read distributions for the overall Top 10 pathogens provided in **Supplementary Data Sheet S1**.

### 3.6 Exploratory ICU-associated stratification

Within the four primary specimen groups, 435/1, 808 orders (24.1%) were classified as ICU-associated services using the department-based proxy. In this exploratory, contextual, proxy-based descriptive stratification, report-interpreted positivity was higher in ICU-associated than non-ICU-associated services for selected specimen types, including blood (148/182, 81.3% [95% CI, 75.0–86.3%] vs 195/291, 67.0% [95% CI, 61.4–72.2%]) and CSF (21/49, 42.9% [95% CI, 30.0–56.7%] vs 42/191, 22.0% [95% CI, 16.7–28.4%]) (**Supplementary Figure 2A**). Mixed detections were also more frequent in ICU-associated services, particularly in BALF (≥2: 168/184, 91.3% [95% CI, 86.3–94.6%] vs 503/690, 72.9% [95% CI, 69.5–76.1%]; ≥3: 136/184, 73.9% [95% CI, 67.1–79.7%] vs 355/690, 51.4% [95% CI, 47.7–55.2%]) and blood (≥2: 90/148, 60.8% [95% CI, 52.8–68.3%] vs 92/195, 47.2% [95% CI, 40.3–54.2%]; ≥3: 50/148, 33.8% [95% CI, 26.7–41.7%] vs 40/195, 20.5% [95% CI, 15.4–26.7%]) (**Figure 5**, ICU panel). These comparisons should be interpreted as exploratory descriptive stratifications based on an order-level care-setting proxy rather than validated patient-level subgroup findings. Tissue subgroup results, particularly for ICU-associated tissue orders, should be interpreted cautiously because of sparse counts.

### 3.7 Exploratory immunocompromised-proxy stratification

Using the diagnosis-text proxy, 67/1, 808 orders (3.7%) were classified as having immunocompromised proxy status. Given the restrictive and proxy-based nature of this subgroup, these findings were treated as exploratory descriptive results rather than a validated clinical subgroup analysis. Summary patterns are provided in **Supplementary Figure 2B**.

## 4 Discussion

In this single-center cohort organized by specimen-specific first eligible orders, we characterized specimen-specific differences in clinical mNGS reporting patterns in hospitalized patients using finalized clinical reports as the analytic anchor. Report-interpreted positivity varied markedly across specimen types, pathogen-group distributions differed across BALF, blood, CSF, and tissue, and mixed detections were common in BALF and blood but uncommon in CSF. These findings are best interpreted as specimen-context-aware differences in routine laboratory reporting outputs rather than as direct evidence of infection status, etiologic attribution, or diagnostic accuracy.

The differences across BALF, blood, and CSF likely reflect both specimen characteristics and clinical context. BALF had the highest positivity and the most frequent mixed detections, consistent with the complex background of respiratory specimens and the broad inpatient settings in which bronchoalveolar lavage is performed. Blood also showed frequent mixed detections, but the interpretive context differs from BALF because positive reports in blood arise in a lower-biomass setting and may be shaped by host and workflow factors. CSF had the lowest positivity and infrequent mixed detections, consistent with the relatively low-background nature of this specimen type. Broad diagnosis-text categories and recorded anti-infective fields likewise indicated different pretest contexts across specimen groups and may partly contribute to variation in reported positivity, pathogen-group patterns, and mixed detections (**Supplementary Tables S5, S6**). These directional differences are broadly consistent with prior specimen-specific studies in respiratory and central nervous system settings (Han et al., 2024; Zhang et al., 2024; Benoit et al., 2024), while prospective clinical metagenomics work has similarly supported the growing role of mNGS in routine infectious disease diagnostics (Fourgeaud et al., 2024). However, our analysis was anchored to finalized clinical reports and was intended to describe cross-specimen reporting patterns under routine laboratory interpretation rather than to evaluate specimen-specific diagnostic performance. In routine practice, mixed detections are more readily interpretable when the organism constellation is concordant with specimen type, syndrome, and host context, whereas in higher-background settings they may also reflect colonization, background signal, or co-detection of uncertain significance. When multiple organisms met reporting criteria, the finalized report reflected organism- and specimen-context-aware sign-out under routine SOPs rather than *post hoc* analytic prioritization; accordingly, multi-organism outputs should be interpreted as report-level co-detections within this reporting framework rather than as ranked etiologic assignments.

Reported DNA-virus detections in blood and CSF warrant particularly cautious interpretation within a finalized-report–anchored framework. In routine clinical practice, these report-level patterns may reflect a mixture of latent persistence, reactivation, transient nucleic acid detection, and, in some cases, clinically relevant active infection, rather than a uniform biological state. Accordingly, prominent report representation of viruses such as Epstein–Barr virus, cytomegalovirus, and herpes simplex virus 1 should not be directly equated with etiologic significance on the basis of mNGS reporting frequency alone. In practice, interpretation of such viral detections requires integration with specimen type, host immune status, syndrome, and orthogonal clinical testing, including targeted PCR and/or serologic results where available.

Our analytical approach also reflects the need to account for laboratory- and run-specific background in clinical metagenomic testing. Reagent and laboratory contamination can materially influence sequence-based outputs, particularly in low-biomass specimens (Salter et al., 2014). In addition to SOP-embedded background handling, prior methodological work has emphasized background filtering and the dependence of apparent signal on laboratory and library characteristics (Du et al., 2022). We did not impose additional research thresholds beyond finalized reports. Instead, we analyzed report-interpreted calls with standardized organism naming to limit *post hoc* reinterpretation.

These results should be read as report-level patterns across specimen types in routine inpatient practice. The unit of analysis was the first eligible order within each specimen category for each patient, and endpoints were anchored to finalized interpretations. The purpose was to describe how positivity, organism distributions, and mixed detections appeared in clinically reported mNGS results, not to infer diagnostic accuracy or causality. Ranking organisms by order-level report presence rather than by reads served the same purpose.

Exploratory ICU-associated stratification added a contextual, proxy-based descriptive layer to these specimen-specific reporting patterns rather than a basis for independent care-setting inference. Compared with non-ICU-associated services, ICU-associated services may differ in patient acuity, specimen mix, sampling intensity, antimicrobial exposure, and pretest probability, all of which could affect report-interpreted positivity and multi-organism outputs. Our ICU definition was based on requesting-department affiliation and should therefore be read as an order-level care-setting proxy rather than a validated patient-location classification; transfers and mixed care settings may also have introduced misclassification. Moreover, ICU-associated differences were not adjusted for specimen mix or other contextual factors and should not be interpreted as independent ICU effects. These ICU-associated patterns are therefore best understood as exploratory descriptive stratifications within routine reporting pathways rather than validated clinical subgroup findings.

Read counts were summarized descriptively and were not treated as direct measures of organism burden. Read magnitude depends on both technical and biological factors, including sequencing depth, host background, library complexity, and pipeline-specific filtering, and its relationship to clinical relevance is not uniform across organisms or specimen types (Greninger, 2018; Simner et al., 2018). We therefore did not apply read-based thresholds or relate read counts to severity or outcomes. RNA-virus analyses were restricted to RNA-tested orders, and DNA-only orders were coded as not tested, avoiding conflation of not tested with negative in a mixed DNA/RNA operational cohort (Greninger, 2018; Simner et al., 2018).

This study has several limitations. It reflects the indications, workflows, background filtering, and reporting rules of a single center and may not generalize directly across institutions or platforms. Some aspects of the observed cross-specimen patterns may nevertheless be more transferable than others: relative differences in report-interpreted positivity, pathogen-group representation, and mixed-detection frequency across major specimen types may reflect broad specimen-context effects that are likely to be observed, at least directionally, beyond this center. In contrast, absolute positivity rates, the report frequency of individual organisms, and the prominence of latency- or reactivation-prone viruses are more likely to depend on platform characteristics, laboratory workflow, local background handling, and center-specific reporting rules. In addition, although endpoints in this study were anchored to finalized clinical reports, a fully auditable and retrospectively reconstructable set of organism- and specimen-specific analytical positivity criteria was not available across the cohort; therefore, reproducibility at the analytical-threshold level and direct inter-institutional comparability of positivity definitions are limited. Conventional microbiology results and treatment-timeline data were not uniformly available, and integrated clinical adjudication incorporating targeted virology or serologic testing was not feasible within the present retrospective design. This limitation is particularly relevant for DNA viruses prone to latency or reactivation, because reported DNA-virus detections could not be further resolved into latent persistence, reactivation, transient low-level nucleic acid detection, or clinically relevant active infection within the present design. Top 10 rankings and specimen-group DNA-virus patterns therefore indicate report frequency within this testing framework rather than direct estimates of causal importance. Because a patient could contribute to more than one specimen-specific cohort, pooled overall summaries are order-based rather than patient-based. ICU-associated and immunocompromised stratifications were proxy-based and exploratory rather than validated clinical subgroup analyses; moreover, ICU-associated differences were not adjusted for specimen mix or other contextual factors and should not be interpreted as independent care-setting effects.

In conclusion, this finalized-report–anchored study showed clear specimen-specific differences in clinical mNGS reporting patterns among hospitalized patients. These results provide a specimen-context-aware reference for interpreting routine clinical mNGS reports, rather than a basis for adjudicating etiologic causation or directly comparing diagnostic accuracy across specimen types.

## Data Availability

The original contributions presented in the study are included in the article/**Supplementary Material**. Further inquiries can be directed to the corresponding authors.
