# Supplementary material for: Specimen-specific differences in clinical metagenomic sequencing reporting patterns in hospitalized patients: a single-center retrospective observational study
Source: Front Cell Infect Microbiol. 2026 May 20;16:1823283. doi: 10.3389/fcimb.2026.1823283 (PMC13231277; doi:10.3389/fcimb.2026.1823283)
Supplement: Supplementary file 1 [file SupplementaryFile1.docx]

**Supplementary Figure Legends**

### This file contains Supplementary Figures S1–S2B.

**Supplementary Figure S1. Mixed-detection frequencies (≥2 and ≥3 pathogens) among mNGS-positive included orders by specimen group, stratified by immunocompromised proxy status**

**
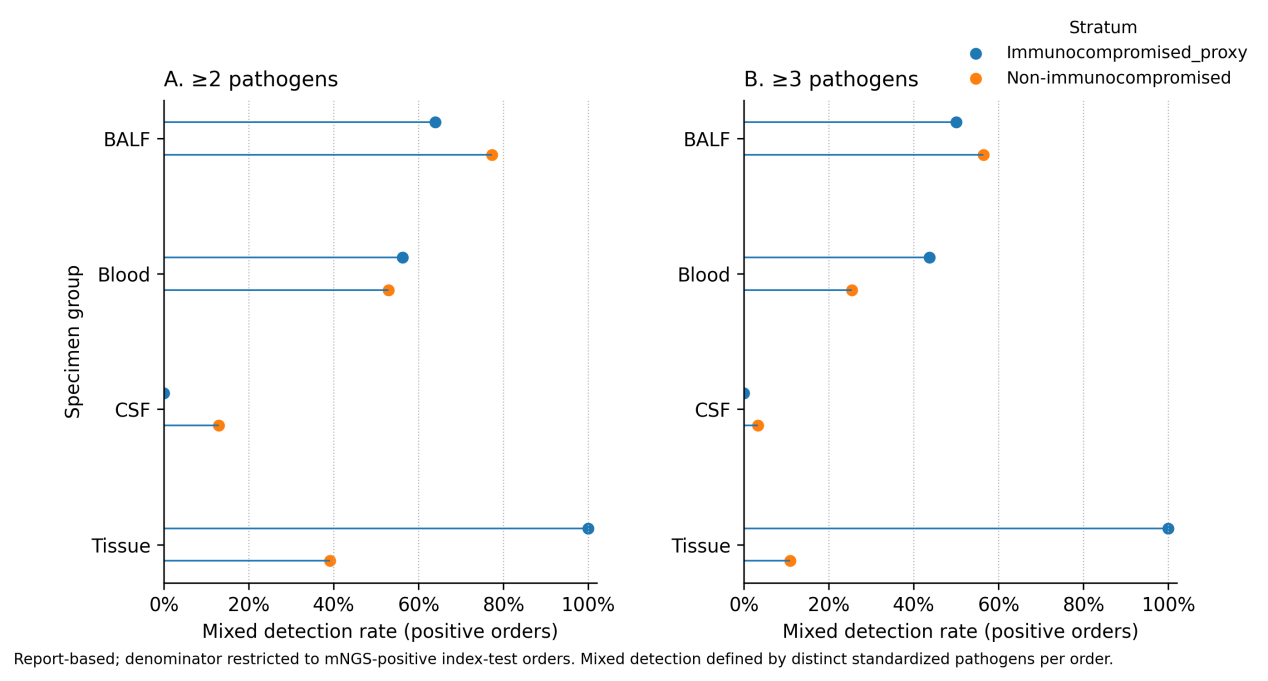
**

**Definitions are the same as in Fig. 5, except stratification is by immunocompromised status derived from a diagnosis-text keyword proxy. This figure is presented as an exploratory proxy-based descriptive stratification only. Percentages were calculated within each specimen × stratum using mNGS-positive included orders (≥1 standardized pathogen detected) as the denominator. Mixed detection was defined as the presence of ≥2 or ≥3 distinct standardized pathogens within the same order. Stratum sizes (mNGS-positive included orders, n) were BALF 14 (immunocompromised proxy) and 862 (non-immunocompromised proxy), blood 13 and 330, CSF 1 and 62, and tissue 1 and 94. Results for very small strata, particularly CSF and tissue orders in the immunocompromised proxy group, should be interpreted cautiously**.

**Supplementary Figure S2A. Any-positive mNGS rate by specimen group stratified by ICU-associated status (requesting-department proxy), 2024–2025**

**
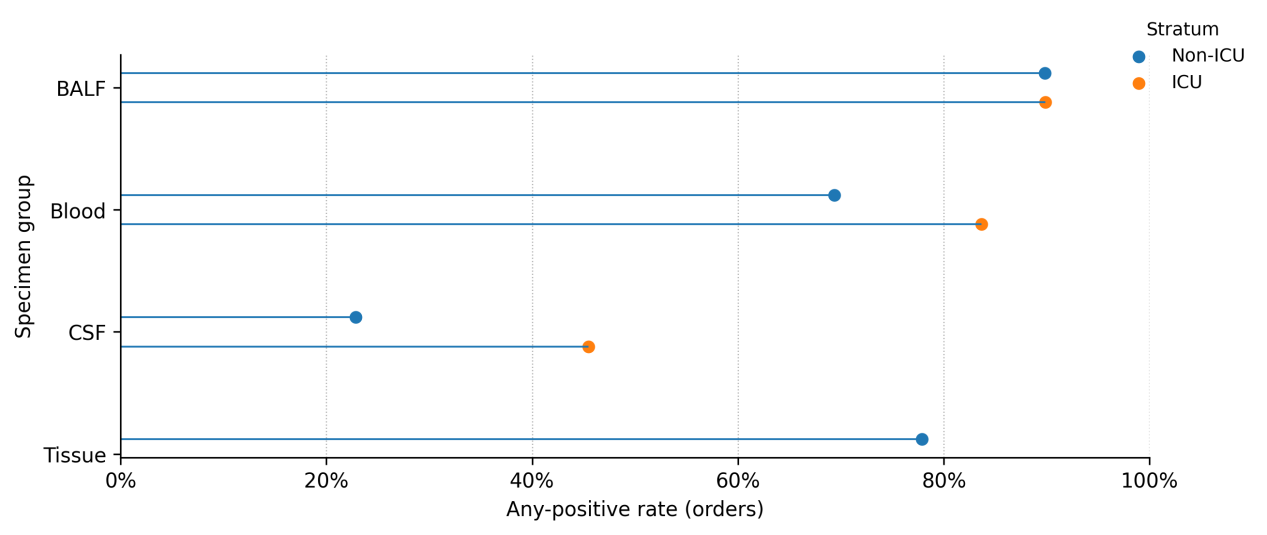
**

**Supplementary Figure S2A. Any-positive rates are shown for included specimen-specific first orders stratified by specimen group (BALF, blood, CSF, and tissue) and ICU-associated status (non-ICU vs ICU), where ICU-associated status was defined using a requesting-department proxy and used as an order-level care-setting attribute. This figure is presented as an exploratory proxy-based descriptive stratification only. Reported percentages use all included orders within each stratum as the denominator. Stratum sizes (any-positive/total orders) were BALF 690/770 (non-ICU) and 186/203 (ICU), blood 195/291 and 148/182, CSF 42/191 and 21/49, and tissue 94/121 and 1/1. Results for very small strata, particularly ICU-associated tissue orders, should be interpreted cautiously.**

### Supplementary Fig S2B. Any-positive mNGS rate by specimen group stratified by immunocompromised proxy status, 2024–2025

**
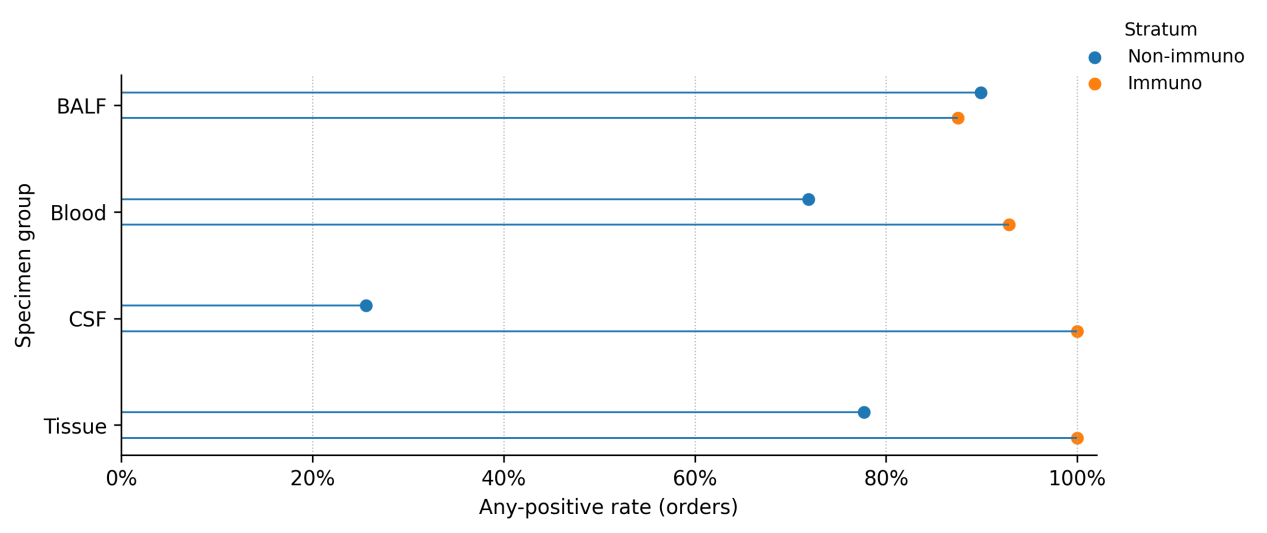
**

Any-positive rates are shown for included specimen-specific first orders stratified by specimen group and immunocompromised status derived from a diagnosis-text keyword proxy. This figure is presented as an exploratory proxy-based descriptive stratification only. Reported percentages use all included orders within each specimen × stratum as the denominator. Stratum sizes (any-positive/total orders) were BALF 862/942 (non-immunocompromised proxy) and 14/31 (immunocompromised proxy), blood 330/444 and 13/29, CSF 62/234 and 1/6, and tissue 94/121 and 1/1. Results for very small immunocompromised-proxy strata, particularly in CSF and tissue, should be interpreted cautiously.
