# Supplementary material for: Specimen-specific differences in clinical metagenomic sequencing reporting patterns in hospitalized patients: a single-center retrospective observational study
Source: Front Cell Infect Microbiol. 2026 May 20;16:1823283. doi: 10.3389/fcimb.2026.1823283 (PMC13231277; doi:10.3389/fcimb.2026.1823283)
Supplement: Supplementary file 2 [file SupplementaryFile2.docx]

**Supplementary Methods**

### S1. Wet-lab workflow overview and specimen preprocessing

Specimens underwent standardized preprocessing prior to nucleic-acid extraction according to the routine PMseq laboratory workflow used during the study period. Pretreatment steps were specimen-type–specific and were designed to improve recovery of microbial nucleic acids, including host depletion and mechanical disruption for selected specimen types.

**Respiratory/pleural-fluid specimens (e.g., sputum/BALF/pleural fluid).** These specimens underwent host depletion and mechanical disruption prior to extraction per routine workflow, including saponin-based host depletion, lysozyme treatment, and bead-beating with 0.5-mm glass beads, followed by DNA extraction.

**CSF and other body fluids.** CSF and other body fluids underwent bead-beating and lysozyme treatment prior to DNA extraction.

**Tissue specimens.** Tissue specimens underwent lysis, bead-beating, and lysozyme treatment prior to DNA extraction.

**RNA workflow enrichment (when PMseq-RNA is performed).** An RNA enrichment step was applied per routine workflow to eligible specimen types, followed by magnetic-bead purification to reduce DNA and increase RNA content. Enriched nucleic acids then underwent fragmentation, reverse transcription, and double-strand synthesis to generate double-stranded DNA for downstream library construction.

### S2. Nucleic-acid extraction, library preparation, and sequencing configuration

**Extraction.** For the DNA workflow, extraction was performed using the routine specimen-appropriate PMseq laboratory workflow used during the study period; for plasma-based DNA extraction where applicable, the TIANamp Micro DNA Kit (DP316; TIANGEN BIOTECH, Beijing, China) was used.

**Library preparation.** DNA and RNA libraries were prepared using the PMseq® Pathogen Sequencing Kit (BGI) following the manufacturer’s standard short-read library preparation protocol. Library quality control was performed using an Agilent 2100 system.

**Sequencing.** Qualified libraries were pooled, converted to DNA nanoballs (DNBs), and sequenced on the MGISEQ-2000 platform (MGI Tech) using single-end short reads (SE75), targeting approximately 80 million reads per sample.

**Mixed DNA/RNA testing.** The cohort included DNA-only orders and PMseq-RNA–tested orders under routine laboratory workflow conditions. In the current analytic dataset, only a small minority of RNA-tested orders were directly identifiable as paired DNA+RNA orders. Accordingly, RNA-virus endpoints were restricted to the subset of orders that underwent PMseq-RNA testing; for DNA-only orders, RNA-virus fields were coded as “not tested” (not equivalent to negative).

### S3. Quality controls and QC-failure handling (endpoint eligibility)

Each run included routine laboratory controls and QC monitoring per SOP, including negative and positive controls and internal control monitoring (as available in routine documentation). QC metrics routinely recorded include sequencing yield, base-quality summaries (e.g., Q30), and internal control performance fields when available.

**QC-trigger handling.** If an order did not meet QC acceptance criteria (e.g., inadequate data yield, poor sequencing quality, or abnormal internal control performance), repeat testing was initiated (re-extraction and/or re-sequencing) per routine workflow. If repeat testing remained nonconforming, repeat sampling was requested when feasible, and the order could remain unresolved for final interpreted reporting or be issued with a QC-related qualification per SOP.

**Binary-endpoint eligibility (analysis rule).** Orders lacking a finalized interpreted positive/negative report outcome (e.g., unresolved QC failure without final sign-out) were excluded from analyses requiring a binary endpoint (any-positive, class presence, mixed detection), and can be summarized descriptively as workflow/QC status.

### S4. Bioinformatic processing and reference databases (functional-level disclosure)

Bioinformatic processing followed the PMseq analytic framework. Reads passing quality filtering were generated after removal of low-quality reads, followed by computational subtraction of human host sequences mapped to the human reference genome hg19 using Burrows–Wheeler Alignment (BWA). Remaining reads were filtered for low-complexity sequences and taxonomically classified by alignment to the Pathogens Metagenomics Database (PMDB), comprising bacterial, fungal, viral, and parasitic references. Reference sequences were obtained from NCBI genomes. Integrated processing and reporting were performed within the HALOS PMseq environment using the PMDB build deployed in routine laboratory use during the study period.

Because detailed algorithmic rule parameters and organism- or specimen-specific analytical positivity thresholds could not be reliably reconstructed across the retrospective cohort, computational processing is described at the functional level: preprocessing, de-hosting, low-complexity filtering, and taxonomic assignment. Analytic endpoints were anchored to finalized interpreted report outputs. Routine read-quality filtering, including adapter and low-quality trimming, was performed within the integrated analytic framework before de-hosting and taxonomic assignment.

RNA-virus handling. RNA-virus detection and analyses were restricted to PMseq-RNA–tested orders; for DNA-only orders, RNA-virus fields were coded as “not tested” and excluded from RNA-virus denominators to avoid misclassification.

### S5. Report interpretation framework (report-based endpoints)

### Report interpretation integrated organism-level signal metrics with specimen context, negative-control/background assessment, and contaminant mitigation per SOP. Organism- and specimen-context–aware reporting rules were applied before final sign-out. Under the routine sign-out workflow, an initial reviewer performed the primary report check and draft interpretation, and a second qualified reviewer performed sequential verification and final sign-out; this was a sequential two-person sign-out workflow rather than a parallel independent review.

Report outputs may present suspected background or commensal organisms separately to support contextual interpretation; these organisms may still be clinically relevant in selected host contexts. For the present study, organisms presented separately for contextual interpretation were not counted as positive organism calls for report-based endpoints unless they were interpreted as positive in the finalized report. Accordingly, between-specimen comparisons reflect final reported outputs under routine clinical interpretation rather than unfiltered analytical detections alone.

Report-based endpoint rule. The binary any-positive endpoint was taken from the finalized interpreted report result, positive or negative, without applying additional post hoc research thresholds. Organism-level analyses used organism calls as presented in the finalized report outputs. A functional summary of the report interpretation framework, endpoint anchoring, and study-period applicability is provided in Supplementary Table S1.

For contextual description only, broad diagnosis-text categories and recorded anti-infective categories were summarized by specimen group and are provided in Supplementary Tables S5 and S6.

### S6. Post-processing for analysis: standardization, class mapping, and denominators

**Name standardization and within-order de-duplication.** Reported organism names were harmonized to standardized English/Latin names using a curated synonym-cluster dictionary (pathogen_name_current → pathogen_name_std). Duplicate entries within an order were defined by order_id + pathogen_name_std and collapsed by retaining the maximum reads value.

**Class mapping.** Standardized pathogens were mapped to prespecified classes using a curated lookup table (pathogen_name_std → class), including bacteria, fungi, DNA viruses, RNA viruses, Mycoplasma/Chlamydia, parasites, and Mycobacterium tuberculosis complex (MTBC; “TB”). The mapping table was harmonized for synonymous labels and is provided for auditability in Supplementary Tables S2–S4.

**Endpoint denominators. All endpoints were calculated at the de-duplicated order level unless otherwise specified:**

**(i) any-positive by specimen: denominator = all orders in that specimen group; numerator = any-positive orders.**

**(ii) class presence by specimen: denominator = all orders in that specimen group; numerator = orders with ≥1 pathogen in that class interpreted as positive in the finalized report (order-level report presence).**

**(iii) Top 10 ranking: pathogens were ranked by order-level report presence, defined as the number of distinct included orders in which the pathogen was reported as positive, rather than by read count.**

**(iv) class composition among positive orders: denominator = de-duplicated pathogen entries among positive orders after within-order de-duplication, with one entry per order per pathogen_name_std.**

**(v) mixed detections: denominator = mNGS-positive orders; numerator = orders with ≥2 or ≥3 distinct standardized pathogens.**

****RNA-virus denominators.** For RNA-virus endpoints, denominators were restricted to PMseq-RNA–tested orders; DNA-only orders were coded as “not tested” and excluded from RNA-virus denominators.**

**Clinical diagnosis-text and recorded anti-infective fields were not used to define analytic endpoints. They were grouped into broad descriptive categories to summarize pretest clinical context (Supplementary Tables S5 and S6)**.

### S7. ICU and immunocompromised proxies: keyword dictionaries, matching rules, and exclusions

**ICU proxy (department-based).** ICU-associated services were identified using prespecified critical-care keywords applied to the requesting-department field. Matching used case-insensitive substring matching and/or regular-expression patterns. Orders were classified as ICU-associated if any ICU keyword matched; otherwise, they were classified as non-ICU-associated.

**Immunocompromised proxy (diagnosis-text–based).** Immunocompromised status was defined using prespecified keyword patterns applied to the diagnosis-text field, covering transplantation, chemotherapy/hematologic malignancy, HIV/AIDS, primary immunodeficiency, immunosuppressive therapy, and prolonged systemic corticosteroid use. Matching used case-insensitive substring matching and/or regular-expression patterns.

**Exclusion patterns and precedence.** Exclusion patterns were applied to reduce false positives (e.g., negation terms or irrelevant contexts). If multiple patterns matched a single record, deterministic precedence rules were applied according to prespecified internal coding rules. These proxies were used for exploratory descriptive stratification only, were not treated as gold standards, and were not intended to support validated clinical subgroup inference or to redefine primary endpoints.
